# Mitochondrial DNA deletion and duplication in Kearns–Sayre Syndrome (KSS) with initial presentation as Pearson Marrow‐Pancreas Syndrome (PMPS): Two case reports in Barranquilla, Colombia

**DOI:** 10.1002/mgg3.1509

**Published:** 2020-10-08

**Authors:** Vanessa Sabella‐Jiménez, Carlos Otero‐Herrera, Carlos Silvera‐Redondo, Pilar Garavito‐Galofre

**Affiliations:** ^1^ Genetics and Molecular Medicine Research Group Universidad del Norte Barranquilla Colombia; ^2^ Genetics, Department of Medicine, Genetics and Molecular Medicine Research Group Universidad del Norte Barranquilla Colombia

## Abstract

**Background:**

Kearns–Sayre Syndrome (KSS) and Pearson Marrow‐Pancreas Syndrome (PMPS) are among the classic phenotypes caused by mitochondrial DNA (mtDNA) deletions. KSS is a rare mitochondrial disease defined by a classic triad of progressive external ophthalmoplegia, atypical pigmentary retinopathy, and onset before 20 years. PMPS presents in the first year of life with bone marrow failure and exocrine pancreatic dysfunction, and can evolve into KSS later in life. Even though an mtDNA deletion is the most frequent mutation in KSS and PMPS, cases of duplications and molecular rearrangements have also been described. In Colombia, few case reports of KSS and PMPS have been published in indexed journals or have been registered in scientific events.

**Methods:**

We discuss clinical and genetic aspects of two case reports of pediatric female patients, with initial clinical diagnosis of PMPS who later evolved into KSS, with confirmatory molecular studies of an mtDNA deletion and an mtDNA duplication.

**Results:**

A large‐scale mtDNA deletion, NC_012920.1:m.8286_14416del, was confirmed by Southern Blot in patient 1. An mtDNA duplication of 7.9 kb was confirmed by MLPA in patient 2.

**Conclusions:**

Our findings are compatible with the phenotypic and genetic presentation of PMPS and KSS. We present the first molecularly confirmed case reports of Colombian patients, diagnosed initially with PMPS, who later evolved to KSS.

## Graphical abstract

We present the first molecularly confirmed case reports of Colombian patients, diagnosed initially with Pearson Marrow‐Pancreas Syndrome, who later evolved to Kearns‐Sayre Syndrome. The first case report is a female patient with a single‐large scale mitochondrial DNA deletion, while the second case report is a female patient with a mitochondrial DNA duplication.

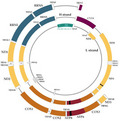

## 1 BACKGROUND

Mitochondrial DNA (mtDNA) deletion syndromes predominantly comprise overlapping phenotypes that may evolve from one clinical syndrome to another in a given individual over time. Kearns–Sayre Syndrome (KSS) and Pearson Marrow‐Pancreas Syndrome (PMPS) are among the classic phenotypes caused by mtDNA deletions. KSS (OMIM #530000) is a rare and sporadic mitochondrial disease with a progressive course (Thajeb, Dai, Chiang, & Shyu, 2006). It is characterized by a classic triad, which consists of onset before the age of 20 years, atypical pigmentary retinopathy, and progressive external ophthalmoplegia. At least one of the minor criteria must be present to contribute to the clinical diagnosis: cardiac conduction block, protein concentration of cerebrospinal fluid (CSF) greater than 100 mg/dl and cerebellar ataxia. Impaired intellect, myopathy in the form of muscle weakness or fatigue, ptosis, failure to thrive, oropharyngeal and esophageal dysfunction (dysphagia), sensorineural hearing loss, nephropathy, or renal impairment, short stature and endocrinopathies are additional clinical features (Goldstein & Falk, 2003).

PMPS (OMIM #557000) classically presents in the first year of life with bone marrow failure and exocrine pancreatic dysfunction. Although the pancreas involvement is specific, it is not present in the majority of PMPS patients (Honzik et al., 2012). Patients have transfusion‐dependent sideroblastic anemia and may be accompanied by thrombocytopenia and neutropenia (Pearson et al., 1979). PMPS may be fatal in infancy without appropriate hematologic management.

The most frequent mutation causing these phenotypes is a single large‐scale mtDNA deletion, ranging in size from 1.1 to 10 kb. The presence of mtDNA deletions in multiple tissues explain the multiplicity of the clinical manifestations. However, cases of duplications and molecular rearrangements have also been described (Goldstein & Falk, 2003). In Colombia, few case reports of patients with KSS and PMPS have been published in indexed journals or have been registered in scientific events. Our case reports are the first molecularly confirmed patients in the country, with initial presentation of PMPS, who later evolved to KSS.

## 2 METHODS

We collected clinical information by review of medical records, as well as evaluation of history, clinical manifestations, laboratory and radiologic findings, and therapy data of two today‐deceased pediatric female patients. Data were reviewed from birth until their deaths at 10‐years‐old and 9‐years‐old, respectively. They were both initially assessed, at 7‐years‐old, at the Genetics clinic after being referred by other specialists. For mtDNA analysis, long‐range PCR study specific for deletion, Southern Blot and multiplex ligation‐dependent probe amplification (MLPA) were performed.

### 2.1 Editorial policies and ethical considerations

Written informed consent for retrospective data collection, molecular studies, and manuscript submission for review and possible publication were obtained from the parents. The study was conducted in accordance with the principles of the Declaration of Helsinki.

## 3 RESULTS

### 3.1 Patient 1

A seven‐year‐oldfemale was referred to the genetics clinic. The patient was the product of the first pregnancy of non‐consanguineous parents. She was born full‐term by cesarean delivery due to stationary labor. Weight at birth was 4000 g and length at birth 53 cm. Mother denied teratogenic drug intake or exposure to mutagenic substances. She had a history of bone marrow aplasia diagnosed during her first year of life. History of pancreatic dysfunction at the time was not documented.

**FIGURE 1:**
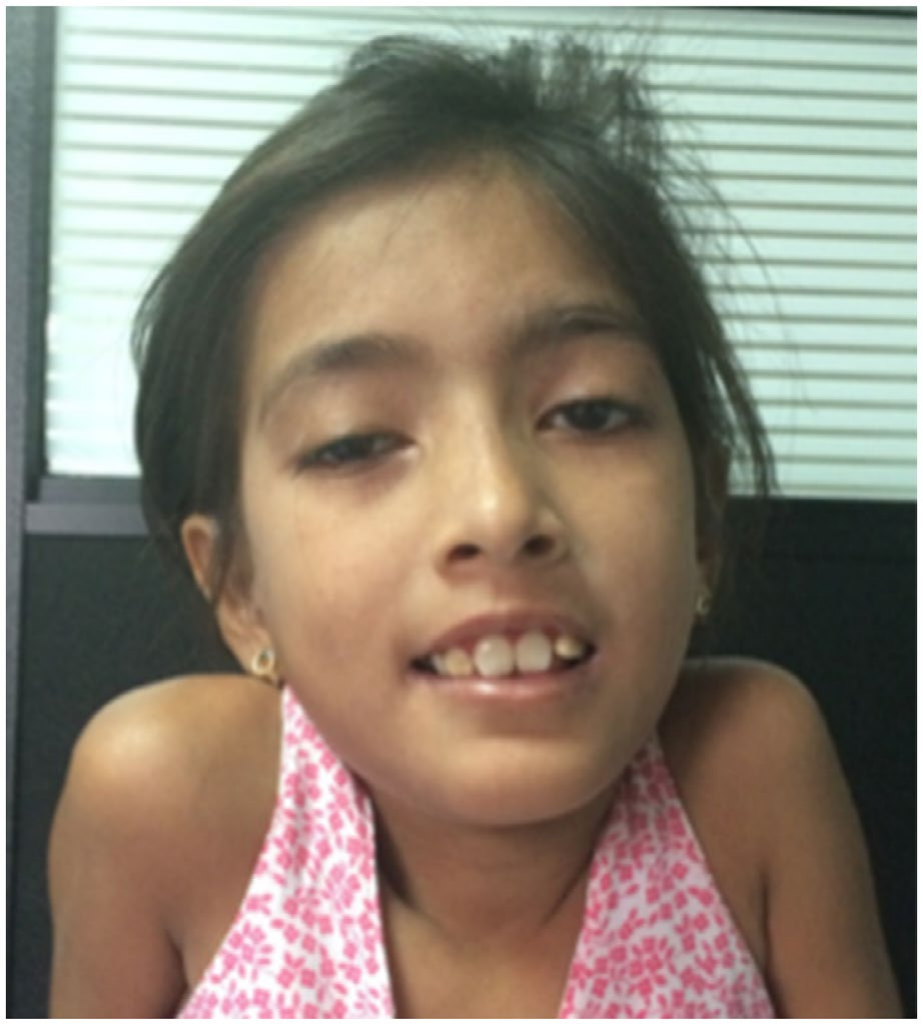
Right palpebral ptosis of patient 1

**FIGURE 2:**
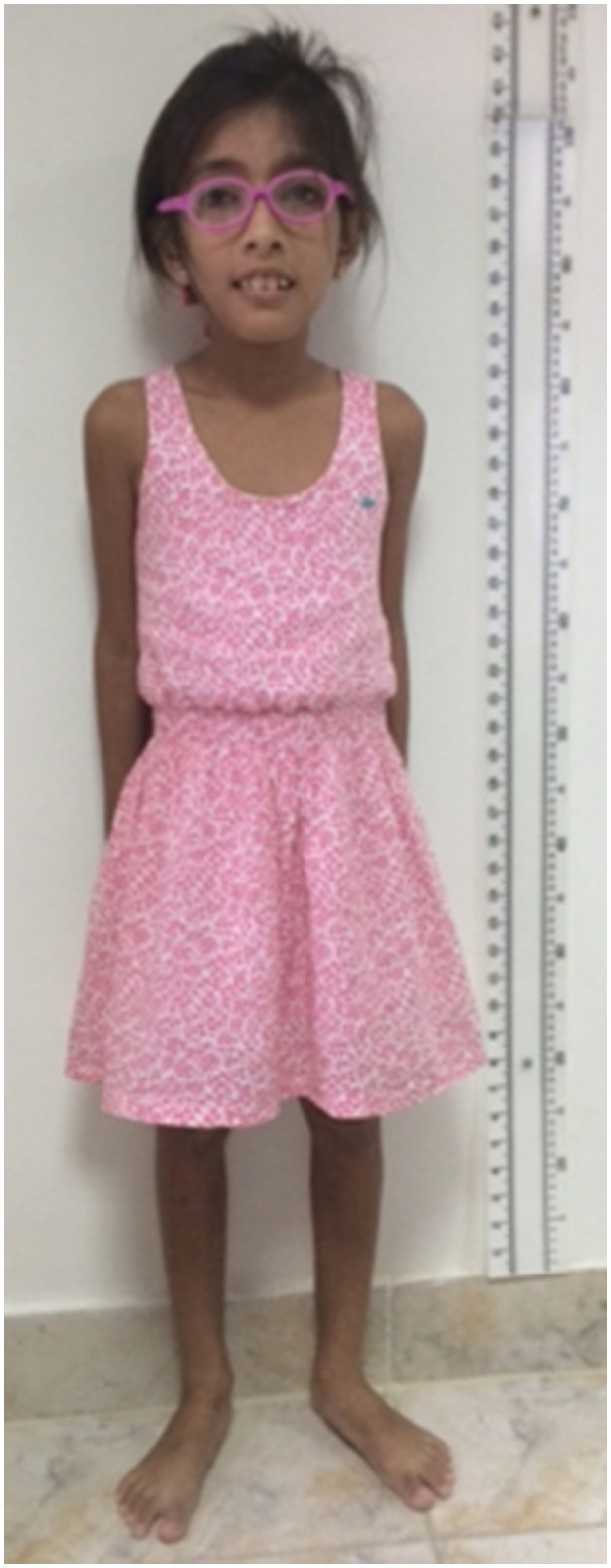
Short stature of patient 1

Physical examination evidenced right palpebral ptosis (Figure 1), external ophthalmoplegia, ocular hypertelorism, decreased visual acuity, prominent incisors, gingival hyperplasia, low‐set ears, sensorineural hearing loss, proximal muscle weakness, hyporeflexia, tremor in the upper extremities, cerebellar ataxia, short stature (Figure 2), and growth retardation.

Laboratory results showed mild pancytopenia in the blood count. Electrocardiogram presented premature ventricular contractions with aberrant conduction. Brain MRI showed severe changes in signal intensity, symmetrically compromising the basal ganglia, cerebral peduncles, and white matter (semioval centers). Ophthalmic fluorescein angiography showed tapetoretinal degeneration and retinitis pigmentosa. Severe bilateral hearing loss was identified in auditory evoked potentials. Lambert test evidenced normal facial nerve. Bone age was 5 years and 1 month. Electromyography of the lower limbs was normal. Esophagogastroduodenoscopy and biopsy showed intestinal mucosal acanthosis and moderate duodenitis.

The long‐range PCR study specific for the deletion detected an mtDNA large‐scale deletion, NC_012920.1:m.8286_14416del, confirmed by Southern Blot (Medical Genetics Laboratories, Baylor College of Medicine). No point mutations or deletions were detected in her parents.

Despite symptomatic and multidisciplinary management, the patient continued with marked deterioration and progressive muscle weakness with subsequent death at 10‐years‐old.

### 3.2 Patient 2

A seven‐year‐olds even female was referred to genetics consult due to suspicion of mitochondrial disease. She was the product of a third pregnancy of non‐consanguineous parents, with a 35‐year‐old mother. She was full‐term born through vaginal delivery. Weight at birth was 3100 g and length at birth was 51 cm. Mother denied teratogenic drug intake or exposure to mutagenic substances. At 3 months of age, she presented severe aplastic anemia requiring ICU care for 10 days. History of pancreatic dysfunction at the time was not documented.

At 2‐years‐old, she was diagnosed with bilateral glaucoma with requirement of surgical intervention. Bilateral palpebral ptosis, external ophthalmoplegia, pigmentary retinitis, decreased visual acuity, and severe amblyopia were diagnosed at 4‐years‐old. She later presented with Fanconi‐type renal tubular acidosis, which was treated with potassium citrate, potassium gluconate, calcium, vitamin D3, zinc, and magnesium by Nephrology. At age 5, she was diagnosed with bilateral severe sensorineural hearing loss and had two cochlear implant placements at 6‐years‐old and at 8‐years‐old. At the age of 7, she presented proximal muscle weakness and cerebellar ataxia, which was treated with coenzyme Q10 and L‐carnitine due to suspicion of mitochondrial disease. An overall and early‐onset neurodevelopmental delay and short stature were observed, with requirement of somatotropin therapy. She was also diagnosed with hypophosphatemic rickets, hypoparathyroidism, and symptomatic hypocalcemia with requirement of phosphate and calcium management. At 8‐years‐old, she had two episodes of focal seizures with right hemiparesis and myalgias of short duration. Levetiracetam was initiated.

Genetics clinic assessment revealed bilateral palpebral ptosis, external ophthalmoplegia, malar hypoplasia, turbinate hypertrophy, tent‐shaped mouth, high‐arched palate, micrognathia, low‐set and winged ears, low‐set hairline, Tanner 1, hirsutism, slight skin pallor, genu valgum, flat‐feet, short stature, generalized hypotonia, hypotrophy, muscle weakness, hyporeflexia, cerebellar ataxia, dyslexic and dysprosodic speech, with alterations in fine motor skills, reading, writing, comprehension, memory, and attention.

Electrocardiogram showed no alterations in heart rhythm and the echocardiogram was normal. Head CT and brain MRI were normal. Electroencephalography (EEG) showed signs of cortical dysfunction and irregular slow activity in the right hemisphere without foci or paroxysms. Control EEG evidenced diffuse slowing in the left frontotemporal region and posterior to the right regions. Her bone age was 7 years, even though her chronological age was 8 years and 10 months at the time.

Within genetic testing, the karyotype showed 46,XX in 25 cells and 533 bands. The study of mtDNA by multiplex ligation‐dependent probe amplification (MLPA) evidenced duplication of 7.9 kb of mtDNA that included the genes *MT*‐*RNR1* (OMIM #561000), *MT*‐*RNR2* (OMIM #561010), *MT*‐*ND1* (OMIM #516000), *MT*‐*ND2* (OMIM #516001), *MT*‐*TC* (OMIM #590020), *MT*‐*TY* (OMIM #590100), *MT*‐*CO1* (OMIM #516030), and part of *MT*‐*CYB* (OMIM #516020) (Gencell Pharma, DNA Alliance). No parental analysis was performed.

Even though the patient continued with multidisciplinary management, she presented with multisystemic complications and subsequent death.

## 4 DISCUSSION

The phenotypes of mtDNA deletion syndromes may evolve from one clinical syndrome to another over time (Goldstein & Falk, 2003). Children, who survive PMPS past the age of 3 years, may progress or develop KSS later in life (Pearson et al., 1979; Rötig, Bourgeron, Chretien, Rustin, & Munnich, 1995) when neurologic and myopathic manifestations appear or worsen. Both patients initially presented with PMPS before and during their first year of life, due to bone marrow failure documentation. Pancreatic insufficiency was not documented in either patient, which reportedly is present in approximately 25% of patients (Tesarova et al., 2019). Patient 1 continued with mild, stable, and non‐transfusion‐dependent pancytopenia, while patient 2 had remission of hematological parameters. Both PMPS‐surviving patients later transitioned to KSS as reported by other studies (Becher, Wills, Noll, Hurko, & Price, 1999; Simonsz, Bärlocher, & Rötig, 1992; Tesarova et al., 2019; Wild, Goldstein, Muraresku, & Ganetzky, 2019).

Even though both patients met the three major criteria and one minor criterion (cerebellar ataxia) of KSS, they were found to have different additional clinical features at the moment of KSS diagnosis. Ocular findings can be observed during the first decade of life (Barrientos et al., 1995). Both patients presented with symmetrical alteration of extrinsic ocular motility, pigmentary retinopathy, and palpebral ptosis, which is usually asymmetric (Goldstein & Falk, 2003). However, patient 2 had bilateral palpebral ptosis. Although the association of KSS and glaucoma seems to be very rare, patient 2 presented glaucoma during early childhood. Open‐angle and normal‐tension glaucoma may be linked to decreased mitochondrial respiratory activity and associated with mitochondrial abnormalities (Abu‐Amero, Morales, & Bosley, 2006; Zarnowski & Kosior‐Jarecka, 2012). Familial primary open‐angle glaucoma (Kalenak & Kolker, 1989), open‐angle glaucoma (Frezzotti & Frezzotti, 2005), normal‐tension glaucoma (Zarnowski, Jaksch, Rejdak, & Zagorski, 2003), and congenital glaucoma (Simaan, Mikati, Touma, & Rötig, 1999) have been reported in individuals with KSS.

Skeletal muscle involvement can manifest as progressive external ophthalmoplegia (PEO), including ptosis and oropharyngeal and esophageal dysfunction (Goldstein & Falk, 2003), altering speech or generating dysphagia. Even though both patients presented with PEO and ptosis, patient 2 had a greater compromise of her speaking skills and ability to swallow. Fatigue and limb muscle weakness, which is more predominantly proximal than distal (Goldstein & Falk, 2003), were features that both patients presented over the later course of the disease.

Cardiac involvement is reported in 50% of cases with KSS. Patient 1 presented with premature ventricular contractions with aberrant conduction, while patient 2 had no documented alteration. Ventricular conduction disturbances such as polymorphic and monomorphic ventricular tachyarrhythmias (Barrera‐Ramirez et al., 2005; Krishna, 2017; Subbiah, Kuchar, & Baron, 2007) have been reported in patients with KSS.

Among central nervous system involvement, both patients had cerebellar ataxia and sensorineural hearing loss. Although the presence of epilepsy is rare in this disease (Goldstein & Falk, 2003), patient 2 had focal seizures. KSS in the setting of epilepsy and long QT syndrome (Berio, Oliaro, & Piazzi, 2010), as well as incomplete KSS with complex partial seizure attacks with secondary generalization (Furuya, Sugimura, Yamada, Hayashi, & Kobayashi, 1997) have been reported. The most commonly reported brain MRI findings in KSS are cerebral and cerebellar atrophy with bilateral high‐T2 signals in subcortical white matter, thalamus, basal ganglia, and brainstem (Meng et al., 2016; Saneto, Friedman, & Shaw, 2008), which correlate with imaging results of patient 1 (bilateral involvement of basal ganglia, cerebral peduncles, and white matter). However, patient 2 had a normal brain MRI.

In KSS, renal findings such as Fanconi's syndrome (Mihai, Catrinoiu, Toringhibel, Stoicescu, & Hancu, 2009; Mochizuki et al., 1996; Mori, Narahara, Ninomiya, Goto, & Nonaka, 1991; Pitchon et al., 2007; Tzoufi et al., 2013) and proximal or distal renal tubular acidosis (Eviatar et al., 1990) have been described among the additional characteristics present. Fanconi‐type proximal renal tubular acidosis was only documented in patient 2, who required electrolyte replacement managements due to increased urinary excretion.

Endocrinopathies are common in KSS, which include hypoparathyroidism and growth hormone deficiency (Goldstein & Falk, 2003). Short stature may be the result of growth hormone deficiency, which both patients presented. However, only patient 2 required somatotropin therapy. Individuals with KSS may also present with hypocalcemia due to hypoparathyroidism (Katsanos, Elisaf, Bairaktari, & Tsianos, 2001), which were additional clinical manifestations present in patient 2, but not in patient 1.

**FIGURE 3:**
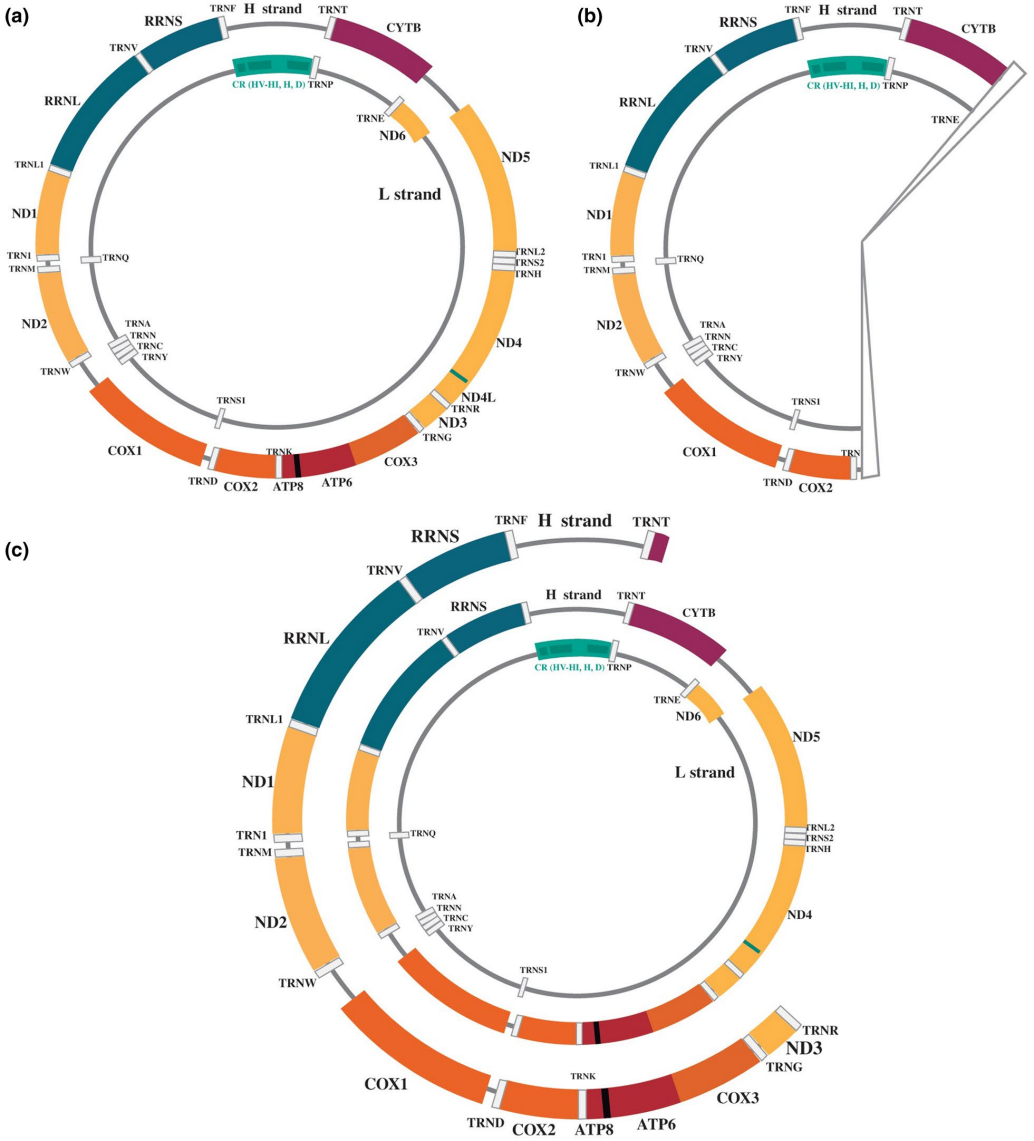
(a) Human mitochondrial genome with heavy (H) and light (L) chains, including 22 transfer RNA genes (white), 2 ribosomal RNA genes (blue), 7 NADH dehydrogenase subunits (yellow), 3 cytochrome c oxidase subunits (orange), 2 ATPase subunits (red) and 1 cytochrome b subunit (purple). In addition, the superposition of the ATP8‐ATP6 and ND4L‐ND4 genes (black), the control region with non‐coding sequence (green) and 3 hypervariable regions (darker green) are shown. (b) Human mitochondrial genome with the mtDNA deletion of the first case presented. (c) Human mitochondrial genome with the mtDNA duplication of the second case presented

In addition, both patients were found to have different mtDNA mutations through genetic testing. The mutation most commonly reported and present in more than a third of patients with KSS, as well as patients with PMPS, is a single large‐scale deletion that may include 50%–75% of mtDNA (DiMauro & Schon, 2003; Maceluch & Niedziela, 2006; Rötig et al., 1995). The most reported deletion, NC_012920.1:m.8470_13446del, involves 4977 bp and 12 mitochondrial genes (Brockington et al., 1995; Goldstein & Falk, 2003). Patient 1 had a single large‐scale mtDNA deletion, NC_012920.1:m.8286_14416del, of 6131 bp and 14 mitochondrial genes involved, similar to the most common reported deletion. (Figure 3b). Associations with more than 150 different mtDNA deletions and KSS have been documented (Goldstein & Falk, 2003) and may include loss of transfer RNA and protein coding genes (Poulton, Morten, Weber, Brown, & Bindoff, 1994), which was the case for patient 1 (Figure 3a,b). Variations in size and types have been observed, with deletions reported from 1.1, 2, 4.5, 7 kb (Zeviani et al., 1988), and up to 10 kb (Poulton et al., 1995). However, the severity of the pathology does not correlate with the size, site, or level of the deletion (Moraes et al., 1989). Reports of patients with KSS, who survived PMPS in infancy, had mtDNA deletions varying from 2.9 to 8 kb (Becher et al., 1999; Simonsz et al., 1992; Wild et al., 2019).

Large‐scale structural rearrangements of the human mitochondrial genome, such as deletion dimers, deletion multimers, and duplications, have been associated with mitochondrial respiratory chain disorders, such as KSS and PMPS (Rötig et al., 1995; Superti‐Furga et al., 1993; Wallace, 1992). Rearranged mtDNA, in the form of deletions and duplications, has been reported in KSS (Kleinle et al., 1997). Large‐scale mtDNA duplications transmitted by maternal inheritance have been described in individuals with KSS (Poulton, Deadman, & Gardiner, 1989), which can coexist with deletions (Brockington, Sweeney, Hammans, Morgan‐Hughes, & Harding, 1993; Cohen & Gold, 2001; Poulton et al., 1994).

Patients who survived PMPS in infancy, later developed KSS and were found to have mtDNA rearrangements in the form of deletions, deletion dimers, and/or duplications (Rötig et al., 1995; Smith, Hann, Woodward, & Brockington, 1995), even in different tissues (Jacobs et al., 2004). Mitotic segregation may explain how patients with PMPS develop KSS in childhood or adolescence. While there is an increase of mtDNA deletions in postmitotic tissues, such as skeletal muscle, there is a decrease of them in rapidly dividing blood cells (Goldstein & Falk, 2003; Larsson, Holme, Kristiansson, Oldfors, & Tulinius, 1990; Rötig et al., 1990). High amounts (20%–95%) of rearranged mtDNA were detected in all tissues from a patient with PMPS and in skeletal muscle from a patient with KSS (Kleinle et al., 1997). Although other studies have found duplications in postmortem tissues in patients with PMPS (Muraki, Sakura, Ueda, Kihara, & Goto, 2001), our case reports lack post mortem studies due to parents' wishes. We only detected a 7.9 kb mtDNA duplication in blood in patient 2 (Figure 3c), similar in size and location as a reported tandem duplication by Brockington et al. (1995).

In conclusion, we observed different additional clinical findings and mtDNA mutations in both pediatric patients. This represents the first molecularly confirmed case reports in Colombian patients, initially diagnosed with PMPS, who later evolved to KSS.

## CONFLICT OF INTEREST

The authors have no conflicts of interests to declare.

## AUTHOR CONTRIBUTIONS

Pilar Garavito‐Galofre and Carlos Silvera‐Redondo assessed both patients in genetics clinic and collected the clinical data of both pediatric female patients. Vanessa Sabella‐Jiménez and Carlos Otero‐Herrera participated in the overall design and drafted the manuscript. Pilar Garavito‐Galofre and Carlos Silvera‐Redondo participated in revising the manuscript. Pilar Garavito‐Galofre is the corresponding author of this manuscript. All authors read and approved the final manuscript.
